# Supplementary material for: Electrostatic and steric effects underlie acetylation-induced changes in ubiquitin structure and function
Source: Nat Commun. 2022 Sep 16;13:5435. doi: 10.1038/s41467-022-33087-1 (PMC9481602; doi:10.1038/s41467-022-33087-1)
Supplement: Supplementary file 1 — Supplementary information [file 41467_2022_33087_MOESM1_ESM.pdf]

Electrostatic and steric effects underlie acetylation-induced changes in ubiquitin structure and function

Kienle et al.

Supplementary Information

SUPPLEMETARY FIGURES

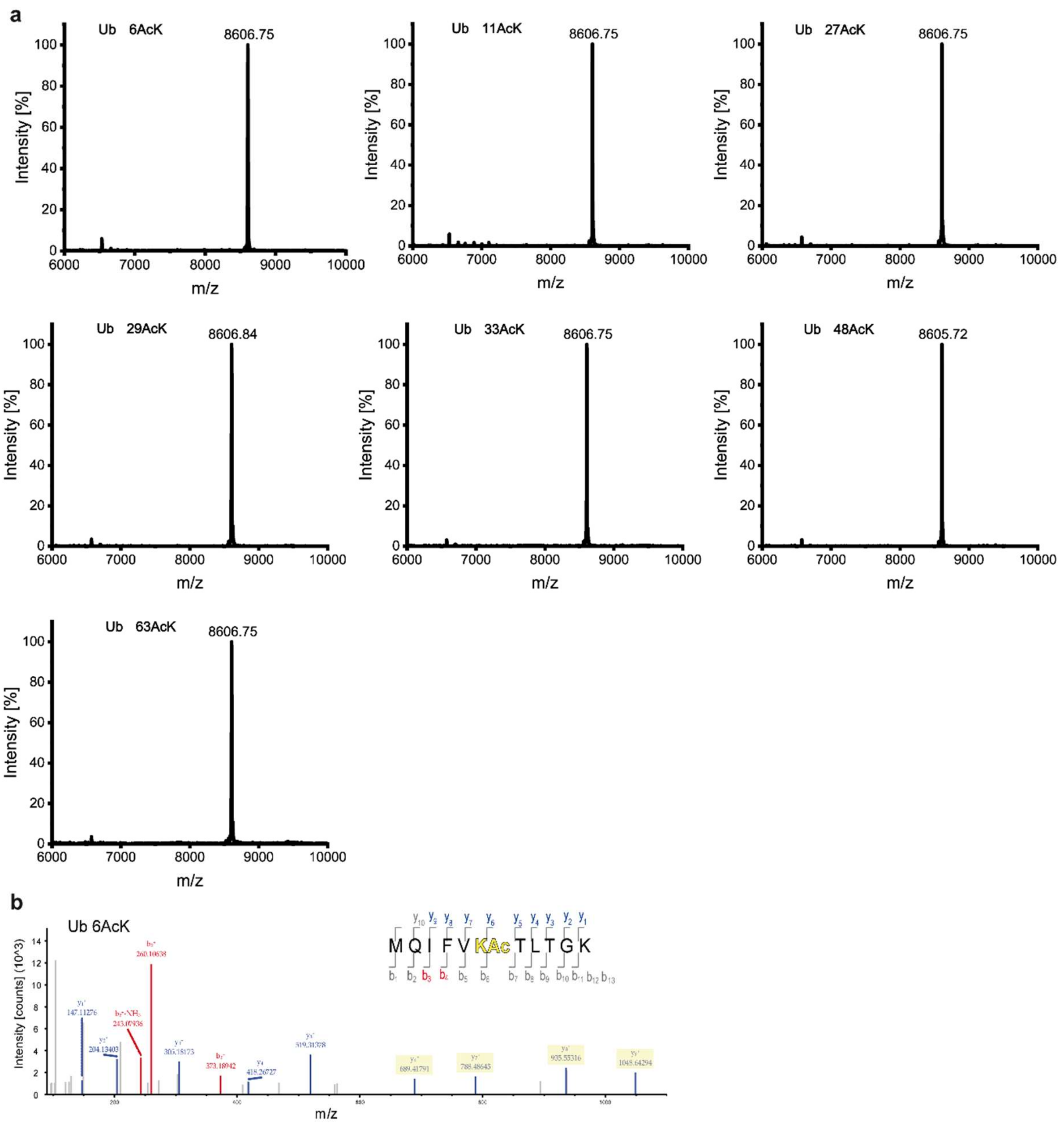

Supplementary Fig. 1. ESI-MS spectra of site-specifically acetylated Ub variants. a, ESI-MS analysis demonstrates the quantitative incorporation of acetyllysine into the different Ub variants. The calculated mass of each acetylated Ub variant is 8606.84 Da. **b,** LC-MS/MS analysis of Ub 6AcK reveals acetylation of K6 (KAc = K + 42 Da). Identified b and y ions are indicated in red and blue, respectively. b ions harboring acetylated K6 are indicated by a light-yellow box.

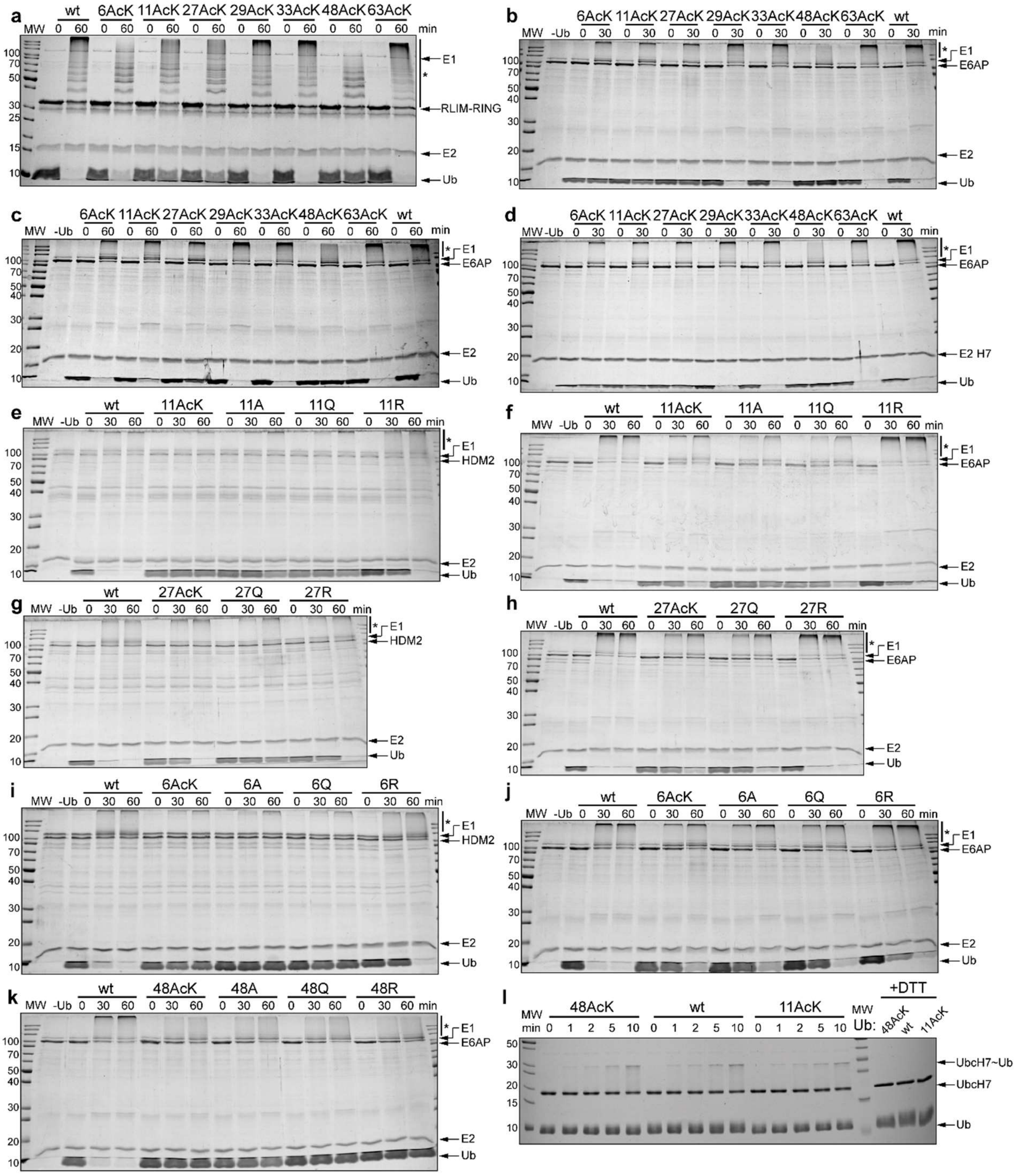

Supplementary Fig. 2. Acetylation of Ub at K6, K11, K27 or K48 interferes with autoubiquitylation of E3s. a-k, Autoubiquitylation reactions were performed as described in Methods and started by addition of the Ub variants indicated or non-modified Ub (wt). Reactions were stopped at the times indicated. Reactions in the absence of Ub (-Ub) were stopped after 30 min ((b) and (d)) and 60 min, respectively. All reactions were analyzed by SDS-PAGE followed by Coomassie blue staining. Running positions of molecular mass markers (MW), UBA1 (E1), UbcH5b (E2), UbcH7 (E2 H7), free Ub (Ub), and the E3 ligases RLIM-RING, E6AP, and HDM2 as well as their autoubiquitylated forms (*) are indicated. **a,** Autoubiquitylation assay with a GST fusion protein of the RING domain of RLIM. **b, c,** Autoubiquitylation assays with E6AP, a member of the HECT family of E3 ligases. **d,** same as **b,** except that reactions were performed in the presence UbcH7 instead of UbcH5b. The results obtained in b and d indicate that the inefficient usage of the Ub variants 6AcK, 11AcK, 27AcK, and 48AcK for autoubiquitylation is not due to the E2 employed but rather to E6AP itself. **e-k,** Autoubiquitylation assays with the Ub AcK variants indicated and Ub variants, in which the respective lysine residue was replaced by A, Q, or R. As E3s, HDM2 (e, g, i) and E6AP (f, h, j, k) were used. **l,**

Thioester complex formation assay of UbcH7 with Ub 48AcK, Ub 11AcK or non-modified Ub (wt). Note that UbcH7 was used, since unlike UbcH5b, it does not catalyze the covalent attachment of Ub to other proteins, allowing to readily detect UbcH7-Ub thioester complexes[34]. Reactions were stopped at the times indicated in the absence of a reducing agent or after 10 min in the presence of a reducing agent (+DTT). Reactions were analyzed by non-reducing SDS-PAGE and Coomassie blue staining. Running positions of molecular mass markers (MW; kDa), free Ub, UbcH7, and the UbcH7-Ub thioester complex are indicated. **a-l,** The experiments shown are representative of three independent experiments. Source data are provided as a Source Data file.

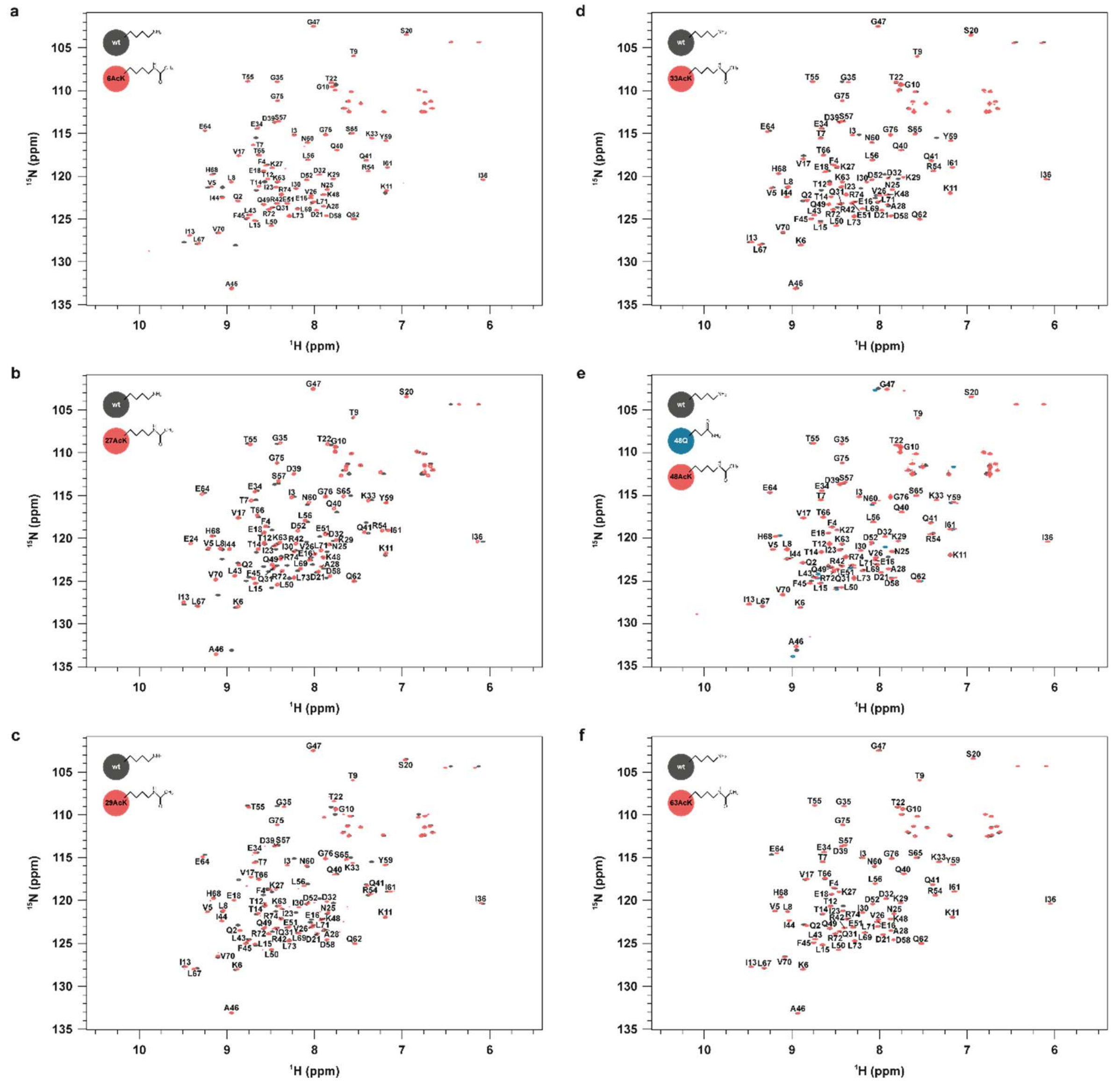

Supplementary Fig. 3. NMR spectra of acetylated Ub variants. a-f, Two-dimensional ^{1}H-^{15}N HSQC NMR spectra of Ub 6AcK (a), Ub 27AcK (b), Ub 29AcK (c), Ub 33AcK (d), Ub 48AcK (e) and Ub 63AcK (f). The corresponding spectrum of Ub 11AcK is shown in Fig. 3a. Resonance signals originating from the acetylated Ub variants are colored in red and are labeled by using the one letter code. A superimposition with the spectrum of non-modified Ub (wt) colored in anthracite is provided from (a) to (f) and the spectrum of Ub 48Q colored in blue is additionally included in (e) for direct comparison. Note that the respective backbone amide nitrogen of the acetylated lysine residues is unlabeled resulting in no cross peak in the respective spectrum. Source data are provided as a Source Data file.

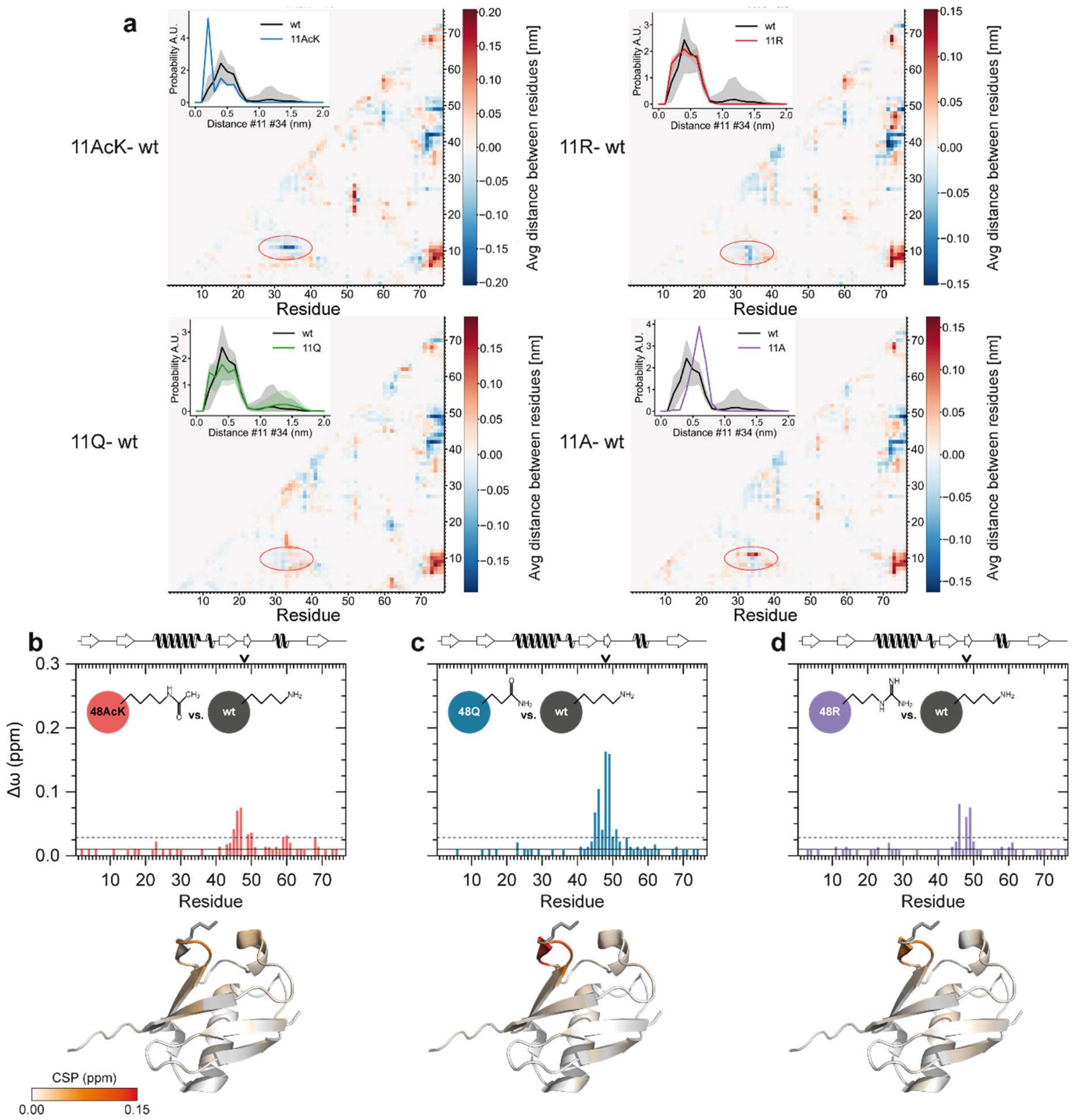

Supplementary Fig. 4. MD simulations illuminating pairwise distance distributions within Ub 11AcK and related Ub K11 variants, and NMR chemical shift perturbation analysis of Ub 48AcK and related Ub K48 variants. a, Impact of different side chains at position 11 on the local environment and the salt bridge with E34. Simulations were performed on Ub 11AcK, 11R, 11Q and 11A. Pairwise minimum distance distributions between residues at positions 11 and 34 are shown for the different Ub variants in comparison to non-modified Ub (wt). The graph at the top left corner illustrates the average value of three independent simulations lasting 1 µs each with confidence bands spanning the extreme values of the individual simulations. The contact maps represent average minimum distances between all residues with respect to non-modified Ub. Thereby, the average distance matrix of non-modified Ub (three independent simulations lasting 1 µs each) is subtracted from the average distance matrix of the respective Ub variant. Distances greater than observed in the simulations of non-modified Ub are indicated in red and shorter distances are indicated in blue. Distances between the 11/34 pair are reduced for 11AcK in comparison to non-modified Ub and to a minor extent for 11R. Larger distances are apparent especially for the 11A variant and less pronounced for the 11Q variant. For 11Q, we found an additional effect regarding the fold of the β1/β2 loop in one of the three simulations causing a strong extension of the distance between the residues at positions 11 and 34. **b-d,** Weighted chemical shift perturbation (CSP, Δω) mappings are shown for

Ub 48AcK (b), Ub 48Q (c), and Ub 48R (d) versus non-modified Ub (wt). The cutoff values are the same for all plots and were calculated by taking the mean (horizontal solid line) and the mean plus one standard deviation (horizontal dashed line), respectively, over all Δω values from (b) to (d) excluding values from the respective residues at position 48. Secondary structural elements according to the NMR solution structure of non-modified Ub (PDB ID 1D3Z)[79] are schematically depicted on the top of the graphs in the upper panel and the same structure is used in the lower panel to illustrate the location and amplitude of the perturbations with colors from white to red in continuous mode. The side chain of K48 is drawn as sticks in gray on the structure and the position in the sequence is indicated by an arrow on top of the graphs. Source data are provided as a Source Data file.

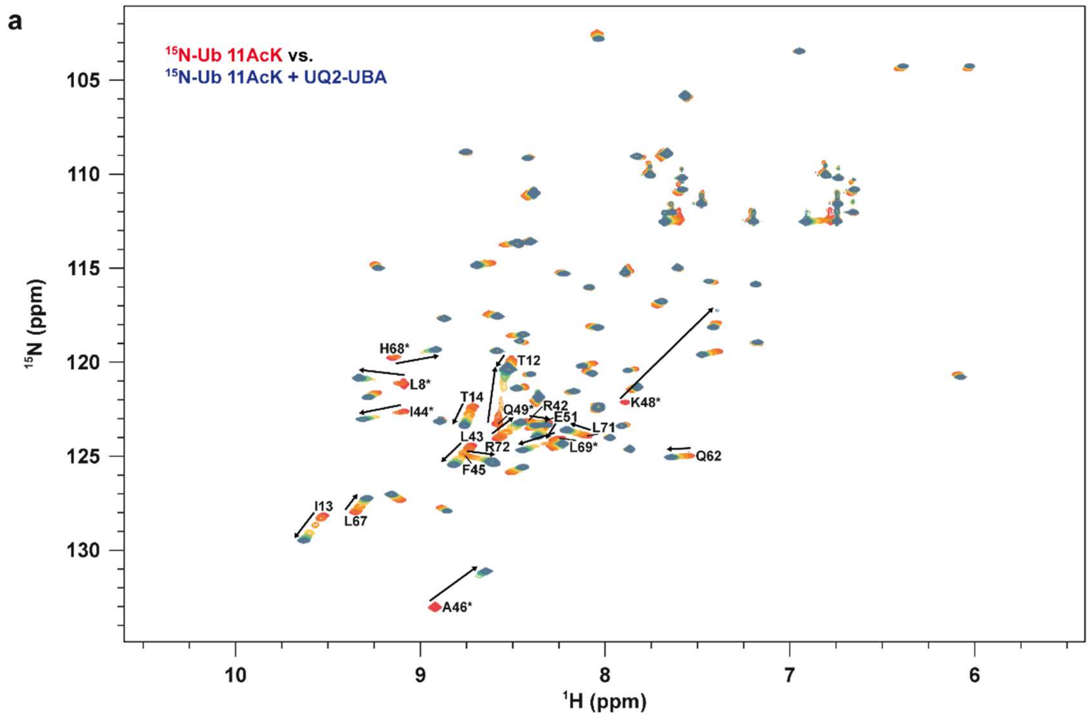

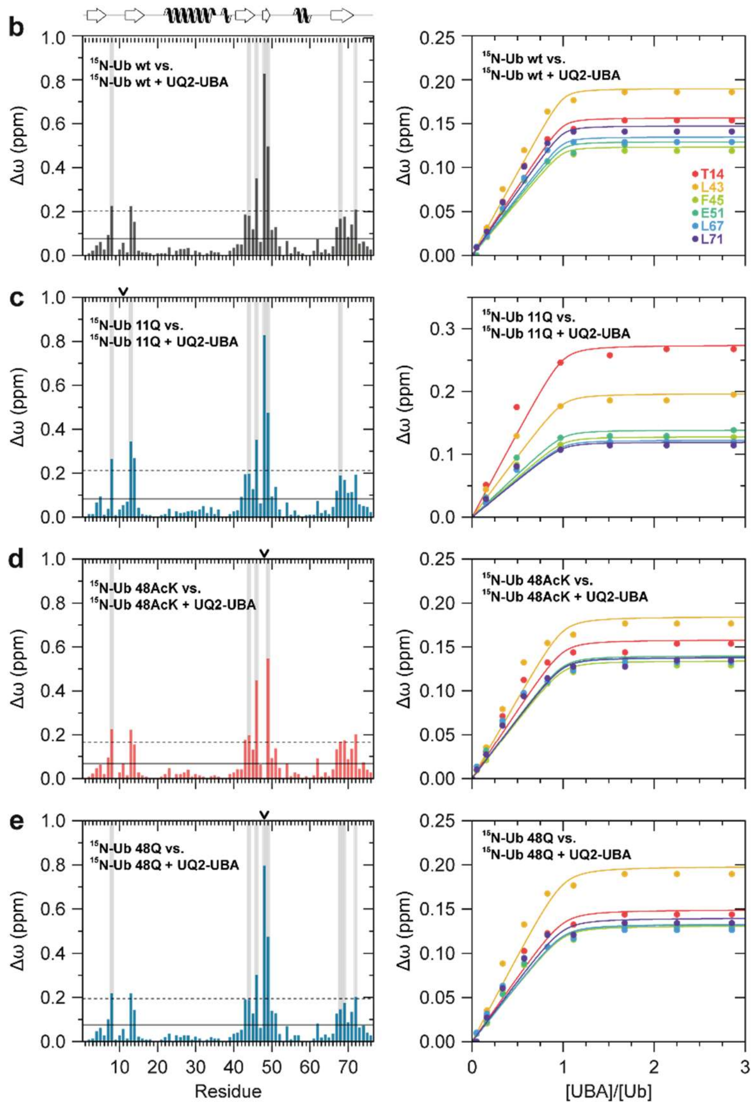

f

Ub variant	*K*d value
Ub wild type	0.5 ± 0.6 μM
Ub 11Q	1.0 ± 1.1 μM
Ub 48AcK	1.3 ± 1.2 μM
Ub 48Q	1.5 ± 1.2 μM

g

Ub variant	*K*d value
Ub wild type	2.0 ± 0.4 μM
Ub 11AcK	8.0 ± 0.8 μM

Supplementary Fig. 5. NMR titration experiments of different Ub variants with the UBA domain of Ubiquilin-2 (UQ2-UBA). a, Superimposition of all two-dimensional ^{1}H-^{15}N HSQC spectra recorded in the NMR titration experiment of ^{15}N isotopically labeled Ub 11AcK with unlabeled UQ2-UBA. The

titration course can be followed by colors changing from red (starting point) to blue (endpoint). Cross peaks of residues that reveal CSP values higher than the mean at the endpoint of the titration experiment (indicated by the horizontal solid line in Fig. 4a) are additionally labeled by the one letter code and their movements in the spectra are indicated by arrows. Residues experiencing more than 90% decrease in signal intensity during the titration course (indicated by gray background lines in Fig. 4a) are marked by an asterisk. **b-e,** Weighted chemical shift perturbation (CSP, $\Delta\omega$) mappings are obtained from NMR titration experiments of ^{15}N isotopically labeled non-modified Ub (wt) (b), Ub 11Q (c), Ub 48AcK (d), and Ub 48Q (e) with unlabeled UQ2-UBA. The values presented were calculated by comparing associated cross peaks in the spectrum at the endpoint and the spectrum at the starting point of the corresponding titrations with each other. CSP values exceeding the horizontal solid line are higher than the mean and values exceeding the horizontal dashed line are higher than the mean plus one standard deviation. Background lines shaded in gray highlight residues experiencing more than 90% decrease in signal intensity during the titration course due to slow or intermediate exchange. Secondary structural elements are depicted on the top of the graph in (b) according to the NMR solution structure of non-modified Ub (PBD ID 1D3Z)[79] and the position of the acetylated lysine residue or of glutamine is indicated by an arrow on the top of the graphs (c-e). Titration curves are shown in the right panel and were derived from the same titration experiments as the corresponding CSP mappings on the left, with a starting concentration of the Ub variants of c = 157 µM. The individual binding isotherms include all residues that were used in the global fitting procedure to determine the respective dissociation constants. **f,** Dissociation constants K_d indicating the binding affinity of the Ub variants for UQ2-UBA as determined by NMR titration experiments with a concentration of the Ub variants at the start of the titration experiment of c = 157 µM. **g,** Dissociation constants K_d indicating the binding affinity of non-modified Ub (wild type) and Ub 11AcK for UQ2-UBA as determined by NMR titration experiments with a concentration of Ub / Ub 11AcK at the start of the titration experiment of c = 33 µM. K_d values shown in (f) are derived from the NMR titration data presented in (b-e); K_d values shown in (g) are derived from the data presented in Fig. 4. Source data are provided as a Source Data file.

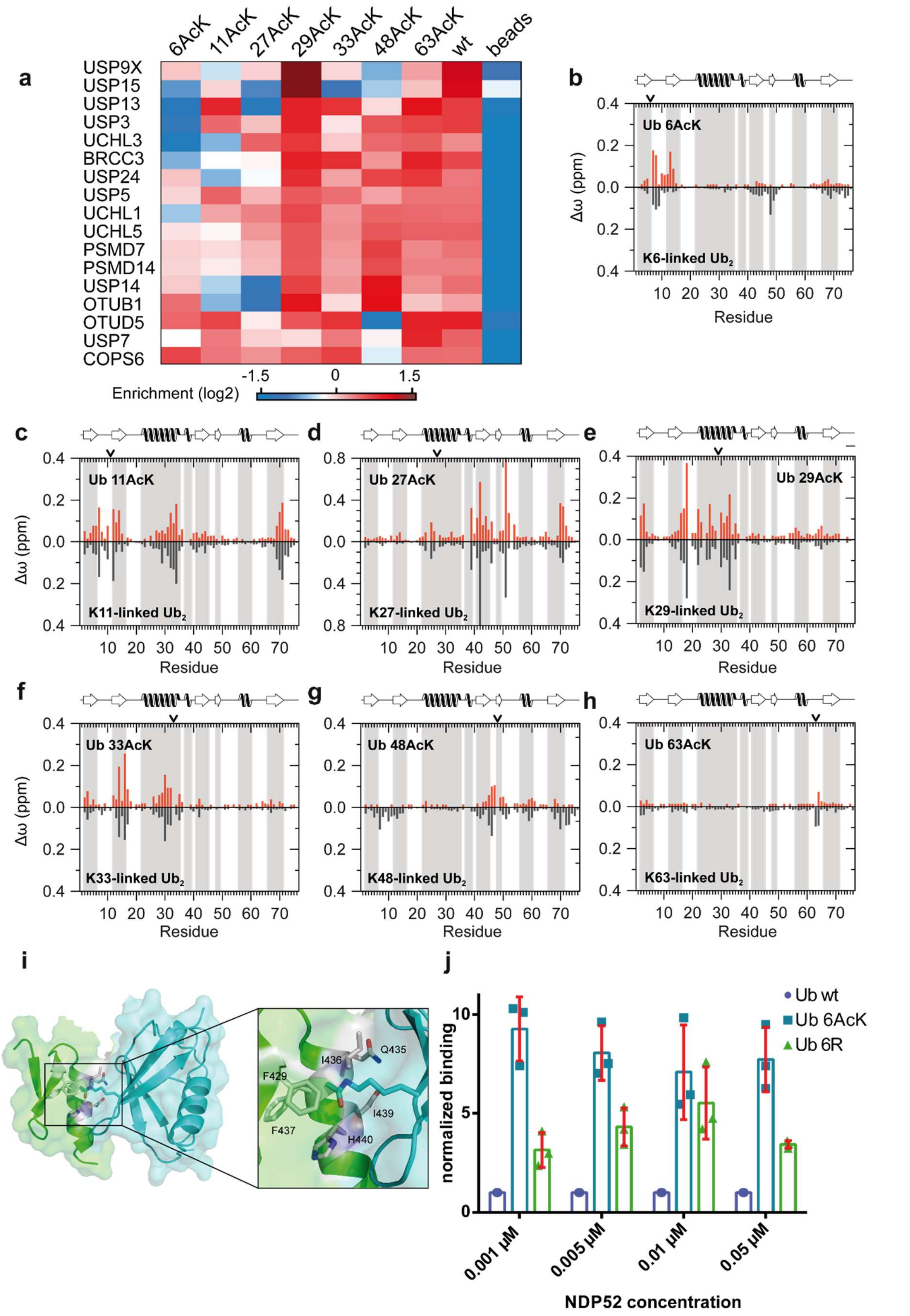

Supplementary Fig. 6. Specific DUBs interact with distinct Ub variants, and Ub acetylation induces chemical shift perturbations similar to those observed for the proximal moiety of the corresponding Ub dimer. a, Distinct binding patterns of DUBs identified by AE-MS for acetylated Ub variants. Red indicates enrichment, whereas blue indicates lack of enrichment. **b-h,** Weighted

chemical shift perturbations (CSP, Δω) calculated for acetylated Ub variants versus non-modified Ub (upper half of the graphs, orange) and for the proximal moiety of the respective isopeptide-linked Ub dimer versus non-modified Ub (lower half of the graphs, gray) are directly compared to each other. Δω values of the Ub dimers were taken from Castañeda *et al.*[47]. In order to achieve comparability, the Δω values of the acetylated Ub variants from our study (see Fig. 2) were recalculated by applying the equation used by Castañeda *et al.*, $\Delta\omega = ((\Delta^1H)^2+(\Delta^{15}N/5)^2)^{1/2}$. Secondary structural elements are depicted on the top of all graphs according to the NMR solution structure of non-modified Ub (PDB ID 1D3Z)[79] and the position of the respectively modified lysine residue is indicated by an arrow. **i,** Crystal structure (PDB ID 4XKL)[12] of the isolated ZF2 domain of NDP52 (green) in complex with in silico (via Vienna-PTM server)[74] acetylated Ub 6AcK (cyan). Subset shows interaction site of K6 of Ub and NDP52 ZF2. Residues F429, Q435, I436, F437, D439, and H440 of NDP52 in gray and in silico acetylated K6 of Ub are shown as stick model. **j,** Increasing concentrations of full-length GST-NDP52 or GST-NDP52 ΔZF2 ("background binding control") were incubated with non-modified Ub (wt Ub), Ub 6AcK, and Ub 6R in an ELISA-like approach. Binding of NDP52 to the Ub variants was monitored by a GST-specific antibody. For calculation of relative binding affinities, values obtained for GST-NDP52 ΔZF2 with the respective Ub variants were subtracted from the corresponding values obtained for GST-NDP52 and normalized to the values obtained with non-modified (wt) Ub (n=3±SD independent experiments). Note that the interaction of the Ub variants with NDP52 is dose-dependent (see Source Data file). Source data are provided as a Source Data file.

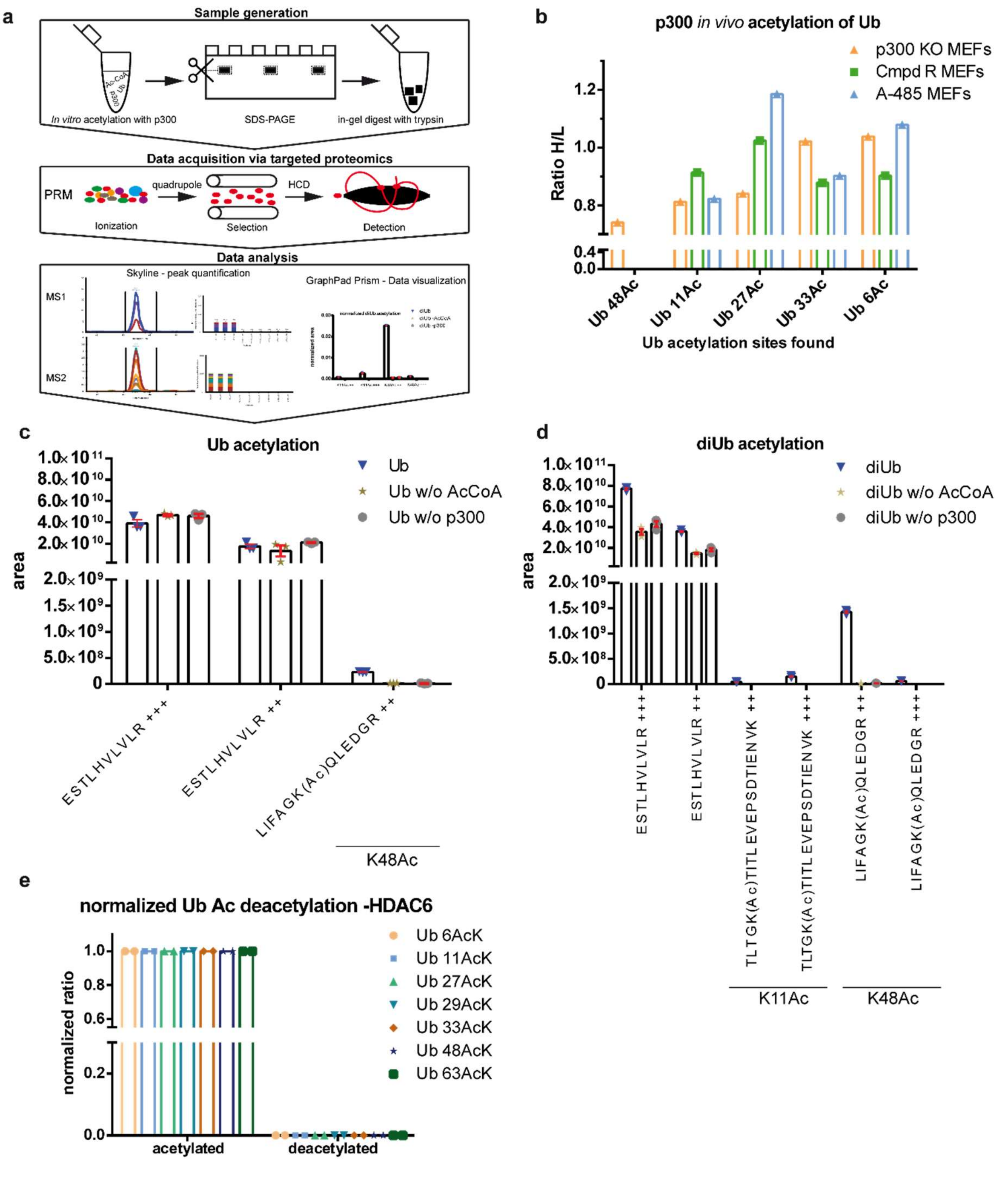

Supplementary Fig. 7. Ub acetylation / deacetylation. a, Workflow used for quantification of p300-mediated acetylation of Ub. **b,** Analysis of the data generated by Weinert *et al.*[21] upon p300/CBP knockout (p300 KO) and by inhibition of acetyltransferase activity of p300/CBP by compound R (Cmpd R) or A-485 (A-485) in mouse embryonic fibroblasts (MEFs). In this report, SILAC-based quantitative MS was applied to identify CBP/p300 regulated acetylation sites. In brief, after cell lysis and sonication, acetone precipitated proteins were digested with trypsin and acetylated peptides were enriched. In each experiment, heavy (H) labels represent respective perturbations and light (L) labels the control. The corrected ratio of H to L (Ratio H/L) is plotted for the respective perturbations and the indicated Ub acetylation sites. Values less than 1 indicate decreased acetylation and values greater than 1 indicate increased acetylation of the respective site upon perturbation. In case of Ub K48 acetylation, acetylated peptides upon treatment with Cmpd R and A-485 were not determined. **c,** Raw values for the peptides used in Fig. 7a to illustrate p300-mediated acetylation of (monomeric) Ub *in*

vitro. Quantified peak areas of the product ions (MS2-level) of the respective precursor with corresponding charge states are shown (n=3±SEM independent experiments). The Ub peptide encompassing residues 64-73 was used for normalization. **d,** Raw values for the peptides used in Fig. 7b to illustrate p300-mediated acetylation of M1-linked Ub dimers *in vitro*. Quantified peak areas of the product ions (MS2-level) of the respective precursor with corresponding charge states are shown (n=3±SEM independent experiments). The Ub peptide encompassing residues 64-73 was used for normalization. **e,** UbAcK variants were incubated in the absence of HDAC6, and reaction products were analyzed by intact protein MS. Peak areas of the respective acetylated/deacetylated Ub variants were used for quantification and normalized to the total Ub peak area. Data were obtained in two independent experiments. Source data are provided as a Source Data file.

SUPPLEMENTARY TABLES

Supplementary Table 1. Oligonucleotides used for cloning.

	No.	Direction (**F**orward **R**everse)	Sequence	
ubiquitin	1	F	TCTTTGTTTAGACCCTGACCGGTAAAACCA	K6-->TAG (codon)
	2	R	ACCGGTCAGGGTCTAAACAAAGATCTGCAT	K6-->TAG (codon)
	3	F	CAGATCTTTGTTAGAACCCTGACCGGTAAA	K6-->R
	4	R	ACCGGTCAGGGTTCTAACAAAGATCTGCAT	K6-->R
	5	F	GCAGATCTTTGTTCAAACCCTGACCGGTAA	K6-->Q
	6	R	ACCGGTCAGGGTTTGAACAAAGATCTGCAT	K6-->Q
	7	F	CGTCTGATTTTTGCAGGTCAACAGCTGGAA	K48-->Q
	8	R	TCTTCCAGCTGTTGACCTGCAAAAATCAGA	K48-->Q
	9	F	CGTCTGATTTTTGCAGGTAGACAGCTGGAA	K48-->R
	10	R	CATCTTCCAGCTGTCTACCTGCAAAAATCA	K48-->R
	11	F	CGTCTGATTTTTGCAGGTTAGCAGCTGGAA	K48-->TAG (codon)
	12	R	ACGACCATCTTCCAGCTGCTAACCTGCAAA	K48-->TAG (codon)
	13	F	CATTCAGTAGGAAAGCACCCTGCATCTGG	K48-->TAG (codon)
	14	R	CCAGATGCAGGGTGCTTTCCTACTGAATG	K63-->TAG (codon)
	15	F	TGTTAAAACCCTGACCGGTTAGACCATTACAC	K11-->TAG (codon)
	16	R	GTGTAATGGTCTAACCGGTCAGGGTTTTAACA	K11-->TAG (codon)
	17	F	ACCGGTCAAACCATTACACTGGAAGTTGAA	K11-->Q
	18	R	TTCAACTTCCAGTGTAATGGTTTGACCGGT	K11-->Q
	19	F	AAAACCCTGACCGGTCGAACCATTACACTG	K11-->R
	20	R	CAGTGTAATGGTTCGACCGGTCAGGGTTTT	K11-->R
	21	F	GAAAATGTGTAGGCCAAAATCCAGGACAAAG	K27-->TAG (codon)
	22	R	CTTTGTCCTGGATTTTGGCCTACACATTTTC	K27-->TAG (codon)
	23	F	ATGTGCAGGCCAAAATCCAGGACAAAGAAGG	K27-->Q
	24	R	CCTTCTTTGTCCTGGATTTTGGCCTGCACAT	K27-->Q
	25	F	GAAAGCCTAGATCCAGGACAAAGAAGGTAT	K29-->TAG (codon)
	26	R	ATACCTTCTTTGTCCTGGATCTAGGCTTTC	K29-->TAG (codon)
	27	F	GCCAAAATCCAGGACTAGGAAGGTATTCC	K33-->TAG (codon)
	28	R	GGAATACCTTCCTAGTCCTGGATTTTGGC	K33-->TAG (codon)
	29	F	AGCGATACCATTGAAAATGTGCGAGCCAAA	K27-->R

	30	R	TTTGGCTCGCACATTTTCAATGGTATCGCT	K27-->R
	31	F	GCAGGTGCACAGCTGGAAGATGGTCGTACCC	K48-->A
	32	R	GGGTACGACCATCTTCCAGCTGTGCACCTGC	K48-->A
	33	F	TCTTTGTTAAAACCCTGACCGGTGCAACCAT	K11-->A
	34	R	ATGGTTGCACCGGTCAGGGTTTTAACAAAGA	K11-->A
	35	F	GCAGATCTTTGTTGCAACCCTGACCGGTAA	K6-->A
	36	R	TTACCGGTCAGGGTTGCAACAAAGATCTGC	K6-->A
AcK-RS (*M.alvus*)	37	F	aatcaattaagctcggcgcgcctgcagg	linearizing pRSF vector for AcK-RS
	38	R	tcacggtcatggtatatctccttattaaagttaaacaaaattatttctacaggggaattgttatccgctcacaattcccc	linearizing pRSF vector for AcK-RS
	39	F	gagatataccatgaccgtgaaatatacc	linearizing AcK-RS (AG Lang) for pRSF vector
	40	R	cgcgccgagcttaattgatttttgcaccattc	linearizing AcK-RS (AG Lang) for pRSF vector
UBA (UQ2)	41	F	ctttcagggcAATCCAGAAGTCAGATTTCAGCAAC	linear sing Uba (UQ2) for pGEX TEV vector
	42	R	tcgtcagtcaGGAGCCCAGCAGCCTTTC	linearizing Uba (UQ2) for pGEX TEV vector
	43	F	gctgggctccTGACTGACGATCTGCCTC	linearizing pGEX TEV vector for Uba (UQ2)
	44	R	cttctggattGCCCTGAAAGTAAAGATTTTCATC	linearizing pGEX TEV vector for Uba (UQ2)
Strep-Ub	45	F	GCAGTTCGAAAAGGGTGCAATGCAGATCTTTGTTAAAACCCTGAC	to generate N-term Strep-Ub
	46	R	GGGTGGCTCCAGCTTGCCATATGTACTGTTTCCTGTGTG	to generate N-term Strep-Ub
NDP52	47	F	ctctctctgaCTGACGATCTGCCTCGCG	linearizing pGEX TEV vector for NDP52
	48	R	tctcctccatGCCCTGAAAGTAAAGATTTTCATCCG	linearizing pGEX TEV vector for NDP52

	49	F	ctttcagggcATGGAGGAGACCATCAAAG	linearizing NDP52 for pGEX TEV vector
	50	R	agatcgtcagTCAGAGAGAGTGGCAGAAC	linearizing NDP52 for pGEX TEV vector
NDP52 ΔZF2	51	F	TAACTGACGATCTGCCTC	NDP52 without ZF2 (stop codon introduction
	52	R	CTGCTGTTGCTCCAAGGT	NDP52 without ZF2 (stop codon introduction
Strep-Ub-G76V-M1-Ub (diUb)	53	F	gcgtggtggtGTTCAGCGTGATCCGAGC	linearizing Strep-Ub wt vector for Ub wt (+ G67-->V mutation)
	54	R	agatttgcatAACACCACGCAGACGCAG	linearizing Strep-Ub wt vector for Ub wt (+ G67-->V mutation)
	55	F	gcgtggtgttATGCAAATCTTCGTCAAAACTCTTACGGGAAAAAC	linearizing Ub wt vector for Strep-Ub wt with G67-->V mutation
	56	R	cacgctgaacACCACCACGCAGGCGCAA	linearizing Ub wt vector for Strep-Ub wt with G67-->V mutation
tRNA (*M.alvus*)	57	F	GGGGGACGGTCCGGCGACCAGCGGGTCTCTAAAACCTAGCCAGCGGGGTTCGACGCCCCGGTCTCTCGCCA	
