# Supplementary material for: Electrostatic and steric effects underlie acetylation-induced changes in ubiquitin structure and function
Source: Nat Commun. 2022 Sep 16;13:5435. doi: 10.1038/s41467-022-33087-1 (PMC9481602; doi:10.1038/s41467-022-33087-1)
Supplement: Supplementary file 3 — Reporting Summary [file 41467_2022_33087_MOESM3_ESM.pdf]

nature portfolio

Corresponding author(s): Andreas Marx, Michael Kovermann, Martin Scheffner

Last updated by author(s): Aug 15, 2022

Reporting Summary

Nature Portfolio wishes to improve the reproducibility of the work that we publish. This form provides structure for consistency and transparency in reporting. For further information on Nature Portfolio policies, see our Editorial Policies and the Editorial Policy Checklist.

Statistics

For all statistical analyses, confirm that the following items are present in the figure legend, table legend, main text, or Methods section.

n/a	Confirmed	
☐	☒	The exact sample size (*n*) for each experimental group/condition, given as a discrete number and unit of measurement
☐	☒	A statement on whether measurements were taken from distinct samples or whether the same sample was measured repeatedly
☐	☒	The statistical test(s) used AND whether they are one- or two-sided<br>*Only common tests should be described solely by name; describe more complex techniques in the Methods section.*
☒	☐	A description of all covariates tested
☐	☒	A description of any assumptions or corrections, such as tests of normality and adjustment for multiple comparisons
☐	☒	A full description of the statistical parameters including central tendency (e.g. means) or other basic estimates (e.g. regression coefficient) AND variation (e.g. standard deviation) or associated estimates of uncertainty (e.g. confidence intervals)
☒	☐	For null hypothesis testing, the test statistic (e.g. *F*, *t*, *r*) with confidence intervals, effect sizes, degrees of freedom and *P* value noted<br>*Give P values as exact values whenever suitable.*
☒	☐	For Bayesian analysis, information on the choice of priors and Markov chain Monte Carlo settings
☒	☐	For hierarchical and complex designs, identification of the appropriate level for tests and full reporting of outcomes
☒	☐	Estimates of effect sizes (e.g. Cohen's *d*, Pearson's *r*), indicating how they were calculated

Our web collection on statistics for biologists contains articles on many of the points above.

Software and code

Policy information about availability of computer code

Data collection	All MS/MS data was collected on an QExcactive HF Hybrid Quadrupole-Orbitrap operated with Tune (version 2.9). MS data were collected on a micrOTOF II (Bruker). NMR experiments were performed at T = 298 K on a Bruker Avance III 600 MHz spectrometer equipped with a TCI cryoprobe or an Avance Neo 800 MHz spectrometer equipped with either TCI triple or QCI cryoprobe. Gel and Western Blot pictures were taken on an Fujifilm LAS-3000 Imager.
Data analysis	MaxQuant (version 1.6.8), Perseus software (version 1.6.10.50), Proteome Discoverer 1.4 , Mascot 2.6, Compass DataAnalysis 4.1 (Bruker), Skyline software 21.1.0.146, NMRPipe (version 10.1), NMRViewJ (version 8.0.a27), GROMACS software suite (version 2018 and 2020; includes LINCS algorithm), Adobe Illustrator CS4, GraphPad Prism 6 (GraphPad Software), AIDA Image Analyzer 4.0 (raytest)

For manuscripts utilizing custom algorithms or software that are central to the research but not yet described in published literature, software must be made available to editors and reviewers. We strongly encourage code deposition in a community repository (e.g. GitHub). See the Nature Portfolio guidelines for submitting code & software for further information.

Data

Policy information about availability of data

All manuscripts must include a data availability statement. This statement should provide the following information, where applicable:

- Accession codes, unique identifiers, or web links for publicly available datasets
- A description of any restrictions on data availability
- For clinical datasets or third party data, please ensure that the statement adheres to our policy

The NMR solution structure and the crystal structure of ubiquitin, the NMR solution structure of lysine free ubiquitin, and the crystal structure of NDP52 ZF2 in complex with ubiquitin used in this study are available in the Protein Data Bank under the accession codes 1D3Z [https://doi.org/10.2210/pdb1D3Z/pdb], 1UBQ

[https://doi.org/10.2210/pdb1UBQ/pdb], 2MI8 [https://doi.org/10.2210/pdb2MI8/pdb], and 4XKL [https://doi.org/10.2210/pdb4XKL/pdb], respectively.
The mass spectrometry proteomics data have been deposited to the ProteomeXchange Consortium (http://proteomecentral.proteomexchange.org) via the PRIDE partner repository with the dataset identifier PXD028797 [https://www.ebi.ac.uk/pride/archive/projects/PXD028797] for the AE-MS data and PXD028813 [https://www.ebi.ac.uk/pride/archive/projects/PXD028813] for the PRM data of p300-mediated Ub acetylation.

Field-specific reporting

Please select the one below that is the best fit for your research. If you are not sure, read the appropriate sections before making your selection.

☒ Life sciences ☐ Behavioural & social sciences ☐ Ecological, evolutionary & environmental sciences

For a reference copy of the document with all sections, see nature.com/documents/nr-reporting-summary-flat.pdf

Life sciences study design

All studies must disclose on these points even when the disclosure is negative.

Sample size

Sample sizes were not predetermined based on statistical methods, but were chosen according to the standards of the field (three independent biological replicates for each condition) and a publication on a closely related topic (Ohtake, F. et al. EMBO Rep. 16, 192-201 (2015)). AE-MS samples were prepared in four biological experiments for all investigated samples, and each of these was measured with technical duplicates.

Data exclusions

No data was excluded, only search criteria applied as described in the mansucript.

Replication

AE-MS samples were prepared in four biological experiments for all investigated samples, and each of these was measured with technical duplicates. Confirmation of selected interactions by Western blot analysis was performed two times.
In vitro ubiquitylation and acetylation experiments were performed at least three times.
Pulldown experiments with recombinant proteins and ELISA-like binding studies were peformed at least three times.
p300-mediated acetylation and HDAC6-mediated deacetylation experiments were performed at least two times.
All attempts at replication were successful.

Randomization

No human or animal subjects were used in the study. Randomization is generally not used in the field and not applicable for the in vitro approaches used. AE-MS samples were measured by members of the Proteomics Center of the University of Konstanz that apart from that were not involved in the respective experiments.

Blinding

n/a. Blinding was not applicable, as for the analysis of the in vitro experiments reaction samples need to be correctly labeled. AE-MS samples were measured by members of the Proteomics Center of the University of Konstanz that apart from that were not involved in the respective experiments. Furthermore, blinding is not used in the field.

Reporting for specific materials, systems and methods

We require information from authors about some types of materials, experimental systems and methods used in many studies. Here, indicate whether each material, system or method listed is relevant to your study. If you are not sure if a list item applies to your research, read the appropriate section before selecting a response.

Materials & experimental systems

n/a	Involved in the study	
☐	☒	Antibodies
☐	☒	Eukaryotic cell lines
☒	☐	Palaeontology and archaeology
☒	☐	Animals and other organisms
☒	☐	Human research participants
☒	☐	Clinical data
☒	☐	Dual use research of concern

Methods

n/a	Involved in the study	
☒	☐	ChIP-seq
☒	☐	Flow cytometry
☒	☐	MRI-based neuroimaging

Antibodies

Antibodies used

Primary antibodies: anti-NDP52 (60732, Cell Signaling Technology), anti-EPS15 (12460, Cell Signaling Technology), anti-HDAC6 (7558, Cell Signaling Technology), anti-VCP (2649, Cell Signaling Technology), anti-USP15 (66310, Cell Signaling Technology), anti-RAD23 ab108592, abcam) anti-USP13 (ab109264, abcam), anti-BRCC3 (ABIN1586883, antibodies-online), anti-ubiquitin (07-375, Merck Millipore), anti-GST (G7781, Sigma-Aldrich)
Secondary antibodies: rabbit (SBA-4050-05, dianova), rabbit (111-035-003, Jackson ImmunoResearch).

Validation

Validation of each primary antibody is available on the manufacturers website under the hyperlinks provided below.
anti-NDP52 [https://www.cellsignal.com/products/primary-antibodies/ndp52-d1e4a-rabbit-mab/60732]
anti-EPS15 [https://www.cellsignal.com/products/primary-antibodies/eps15-d3k8r-rabbit-mab/12460]

anti-HDAC6 [https://www.cellsignal.com/products/primary-antibodies/hdac6-d2e5-rabbit-mab/7558]
anti-VCP [https://www.cellsignal.com/products/primary-antibodies/vcp-7f3-rabbit-mab/2649]
anti-USP15 [https://www.cellsignal.com/products/primary-antibodies/usp15-d1k6s-rabbit-mab/66310]
anti-RAD23 [https://www.abcam.com/hhr23a-antibody-epr4818-ab108592.html]
anti-USP13 [https://www.abcam.com/usp13-antibody-epr4348-ab109264.html]
anti-BRCC3 [https://www.antibodies-online.com/antibody/1031281/anti-BRCA1+BRCA2-Containing+Complex,+Subunit+3+BRCC3+N-Term+antibody/?sku=1586883]
anti-ubiquitin [https://www.merckmillipore.com/DE/de/product/Anti-Ubiquitin-Antibody,MM_NF-07-375]
anti-GST [https://www.sigmaaldrich.com/US/en/product/sigma/g7781]

Eukaryotic cell lines

Policy information about cell lines

Cell line source(s)	ATCC (HEK293T)
Authentication	The cell line was not authenticated.
Mycoplasma contamination	Cell line was not tested for Mycoplasma contamination.
Commonly misidentified lines (See ICLAC register)	No commonly misidentified cell line was used in this study.
